# Basic Values Transform Political Interest into Diverse Political Values, Attitudes and Behaviors

**DOI:** 10.1007/s10964-022-01654-w

**Published:** 2022-07-08

**Authors:** Håkan Stattin, Erik Amnå

**Affiliations:** 1 Department of Psychology, Box 1225, Uppsala University, SE-75142 Uppsala, Sweden; 2 Örebro University, School of Humanities, Education and Social Sciences, SE-70182 Örebro, Sweden

## Abstract

Since politically interested adolescents do not necessarily present humanistic, environmental and democratic values, this study addresses the hitherto ignored role that how these basic human values play in politically interested adolescents’ political values, attitudes and behaviors. A cluster analysis of 857 Swedish upper-secondary students (50.8% girls, M_age_ = 16.62, *SD* = 0.71) identified politically interested adolescents who attached high levels of importance to others’ welfare and politically interested adolescents who attached low levels of importance. They differed on most comparative measures: environmental values, inclusive attitudes towards immigrants, support of democratic principles, trust in social movements, and readiness to step in if something jeopardizes the welfare of others. The conclusion is that the value of attaching high importance to others’ welfare or not transforms youth’s political interest into diverse attitudes and behaviors. The cluster group of politically interested adolescents who attached low importance to others’ welfare largely consisted of males.

## Introduction

Political interest is probably the most important predictor of many different aspects of political engagement (Brady, Verba & Schlozman, 1995; Prior, 2019; Shani, 2009). Political scientists, media and others seem pleased when the political interest of youth is high, with the often-implicit assumption that this is good for democracy and the development of society. However, they seem to turn a blind eye to, for example, extremists also working on the basis of political interest but attaching less importance to others’ welfare as a driving force. The role that basic human values play in politically interested youth’s political attitudes and behaviors has been largely ignored in the literature. In this study, politically interested adolescents who attach high levels of importance to the welfare of other people are compared with adolescents who attach low levels of importance to it. In person-oriented analyses, it was expected that these two groups would emerge as naturally occurring clusters. The study examines the evidence that these two groups of adolescents differ on other basic values, attitudes and behaviors, and specifically on their political actions and the perceived need to act when others are in jeopardy.

The best known theory of human values is Shalom Schwartz’s theory of basic human values (Schwartz, (1994); Schwartz, (2016); Schwartz et al., 2012), where he argues that values express people’s desired goals and organizing principles for their behavior, and guide their attitudes and activities in everyday life. His theory contains ten motivational values that are thought to differentiate between people world-wide. One is universalism, consisting of people’s societal concerns, e.g., protection of the welfare of all people, and their wish to protect nature. Jointly, universalism and benevolence, i.e., caring for the welfare of ingroup members, represent the self-transcending value of caring for others. Of the ten values in Schwartz’s theory (self-direction, stimulation, hedonism, achievement, power, security, conformity, tradition, benevolence, universalism), universalism and benevolence are often among the values most highly reported by adults (Schwartz & Bardi, 2001), and even among 7 to 11-year-old children (Döring et al., 2015). One of the components of the universalism values, societal concern, is the focus here. This value, defined here as attaching importance to other people’s welfare, will in this study be analyzed simultaneously with the political interest of adolescents. This will essentially involve comparison between highly politically interested adolescents who attach high levels of importance to the welfare of other people and highly politically interested adolescents who attach low levels of importance to it. Because these two groups are presumed to differ regarding their views on the importance of the welfare of others, they can be expected also to differ on other values, attitudes and behaviors.

In Schwartz’s model (Schwartz, et al., 2012), the importance attached to the welfare of other people is intimately connected with the value of preserving the environment; these values together make up the broader universalism value. Hence, people who embrace equality, justice and helping other people also tend to respect and protect nature. This is likely to be the case for adolescents with a high political interest as well. If politically interested adolescents attach high importance to the welfare of others, they are also likely to endorse preserving the environment. Further, if adolescents’ environmental values are organizing principles for their behaviors, then these values should guide their environmental activities and thinking in everyday life (Bardi & Schwartz, 2003; Schwartz & Butenko, 2014). Highly politically interested adolescents who attach high importance to others’ welfare, compared with politically interested adolescents who attach low importance, should be more likely, for example, to practice pro-environmental behaviors in daily life and feel that they have a strong collective environmental efficacy, i.e., the perception of being capable of coping with the threat of climate change together with others (Jugert et al., 2016).

Attaching high importance to others’ welfare among politically interested adolescents can underly politically interested adolescents’ broader support for the norms of democratic citizenship. For example, social justice and equality are key characteristics of the value of societal concern in Schwarz’s value model. They are also basic democratic principles (Miller, 1978). This study examines whether having positive attitudes towards democratic principles are higher among politically interested adolescents who attach high importance to others’ welfare than among those who express low importance to others’ welfare. Also, trust in social movements built on human rights, the environment or animal rights may be higher among politically interested adolescents who attach high importance to others’ welfare. Further, attaching importance to others’ welfare may be associated with an inclusive attitude towards minority groups. Substantial associations between universalism values and an inclusive attitude towards minority groups have been reported earlier (Kuntz, et al., 2015; Sagiv & Schwartz, 1995; Vecchione et al., 2012). For example, Vecchione & coworkers (2012) found that the universalism value affected adults’ perceptions that immigration has positive consequences. The present study compares adolescents’ inclusive attitudes towards immigrants between politically interested adolescents with high and low levels of values regarding others’ welfare. In short, these two groups of highly politically interested adolescents may show very different values, attitudes and behaviors in relation to the environment and support for democratic norms: democracy and human rights, trust in social movements, and inclusivity regarding immigrants. Thus, politically interested adolescents are not assumed to be a homogenous group. They encompass individuals whose belief systems are different. Accordingly, political interest per se may be an important but insufficient indicator of the core welfare and democratic positions of adolescents.

### Political Participation

How do politically interested adolescents who attach high or low importance to others’ welfare, channel their interest in politics into political action? A lot is known about political participation (Brady, 1999, p. 737). Why youth engage in civic or political activities has been widely examined, based on the roles played by many factors – age, heritage, socio-economic resources like education, gender, cognition, personality, emotions, traits, recruitment, networks, life experiences, and political motivations (Giugni & Grasso, 2022). But they have not been addressed in terms of the motivational role that basic human values play (Deutsch, 2022). This is surprising, because a match would be expected between youth’s care of fellow citizens and their engagement in civic and political activities, which usually have the same value motivation.

Adolescents can decide to engage in politics. Their choice to participate should depend on the type of political activities. Some adolescents engage in civic activities together with likeminded people. Here, they are able to act as young active citizens to affect political processes that they value highly. In the Swedish political milleu, politically interested adolescents who attach high importance to others’ welfare, the environment, and democratic principles, and who are inclusive of minorities, should have more options to participate in politics with others than other adolescents due to their values congruence with the wider political context (Grasso & Giugni, 2016). Other adolescents decide to become formal members of civic or political associations or engage in community services. When adolescents in community services report what they have learned, they mention developing stronger responsibilities for fellow citizens, having obligations to others and the common good, being aware of social inequalities in society, and having a sense of themselves as part of the public (Flanagan, 2013). A focus on citizen welfare should characterize both politically interested adolescents with high levels of importance attached to other people’s welfare and the stated purposes of most civic and political associations. Hence, the politically interested adolescents who attach high importance to others’ welfare would be expected to be more involved in civic actions with others or in political or civic associations than the politically interested adolescents who attach low importance to others’ welfare.

Joining associations or participating in joint activities may not be the preferred or most common way for adolescents to express their political opinions. Adolescents have other means of expression, e.g., donating to preferred political/civic initiatives, wearing a bracelet, badge or other symbol to show support for a good cause, or refusing to buy products for political, ethical or environmental reasons (Ekman & Amnå, 2022). Hence, they can act independently. Also, it is expected that these individual modes of expressions will be more common among politically interested adolescents who attach high importance to others’ welfare.

Whether this also applies to political activities on the internet is an open question. Here, adolescents may choose to interact with people with whom they are familiar or unfamiliar (Minozzi et al., 2020). They can also use the internet and social media by following political and societal information solitarily. This study examines whether the two groups of adolescents differ in these regards – interacting politically on social media with friends or unknown persons, or searching solitarily for political and societal information. Overall, this study has gone some way to identifying potential ways in which politically interested adolescents who attach high importance to others’ welfare can channel their political interest into political action offline and online differently from politically interested adolescents who attach low importance to others’ welfare.

Finally, the question about politically interested adolescents’ political participation brings up the often-discussed issue of how active citizens should be. In their seminal study, Almond & Verba, (1963) compared various political systems in their search for the prerequisites of a stable liberal democracy. One of their main arguments was for a civic culture of shared social norms where citizens were not fully but sufficiently active in politics, and allegiant enough not to jeopardize effective governance. In other words, citizen participation was more a latency than a reality, and indeed – over the past half century since then – citizens in mature democracies have become even less allegiant (Dalton & Shin, 2014).

From the present point of view, if there are youth who are ready to act politically if something bad happens to other people, these are likely to be the ones who are fueled by both political interest and attaching high importance to other’s welfare. They are the ones who should be willing to act politically if people are in jeopardy or are treated unfairly, or just if other people ask them.

That democracies need people who are knowledgeable and can monitor the consequences of political actions is a common idea (Flanagan, 2013; Schudson, 1998). The term standby citizen was coined for participatory latency, which includes a readiness to step in (Amnå & Ekman, 2014; Jennings & Stoker, 2016). Standby citizens should constitute a societal asset since their values make people vigilant, and easily trigger their macro concern that other people may be treated unjustly (Boehnke & Schwartz, 1997; Schwartz, Sagiv, & Boehnke, 2000); like watchdogs, they should be alert when other people’s well-being is jeopardized. The hypothesis is that youth who are politically interested and attach high importance to others’ welfare constitute the adolescents who, in particular, should be likely to intervene and manifest their values if they regard others’ welfare is at risk.

### Gender Differences

The broad value of universalism, encompassing societal concern and care for the environment, has often been found to characterize females more than males. The most robust examination of gender differences in universalism was reported by Schwartz & Rubel, (2005). Their study covered 127 adult samples in 70 countries and involved more than 77,000 persons. They found that females scored higher than men on the value of universalism in over 80% of these samples. This gender difference has also been reported for young adults (Ardenghi et al., 2021) and for 7 to 11-year-old children (Döring et al., 2015). Outside Schwartz’s theoretical framework, review studies conclude that females tend to express more concern, and report stronger environmental attitudes and behaviors than men (Gifford & Nilsson, 2014; Zelenzny, Chua, & Aldrich, 2000).

Based on these findings, it can be expected in this study that girls will attach higher levels of importance to others’ welfare and preserving the environment than boys. Assuming that there are small gender differences in political interest, the cluster of highly politically interested adolescents who attach high levels of importance to others’ welfare then can be expected to consist of more girls than boys. How this translates into gender differences in political values and activities is unclear. Potentially, females’ gender socialization, stronger other orientation and social responsibility (Zelenzny et al., 2000) can be connected to higher levels of support for democratic citizen norms than males´.

## Current Study

Even if adolescents’ political interest is perhaps the most central aspect of their political development (Prior, 2019), a political interest may not be enough to activate young people politically. This study, guided by Schwartz & colleagues,’ (2012) theory of basic human values, contributes to the political socialization literature by suggesting that politically interested adolescents who attach high importance to others’ welfare have very different values, attitudes, behaviors, political actions, and intentions to stand-by for others at risk than their counterparts who attach low importance to others’ welfare. The role that universalism values have in citizens’ ideology, political activism, political values and voting has been examined previously (Caprara et al., 2017). However, no previous study has had the purpose of determining the role that universalism plays in the values, attitudes and political activities of the potentially most important group of adolescents—those with a high interest in politics. Further, confirmation of the hypotheses in the study would raise the question of whether civic knowledge and political visibility differ between the politically interested adolescents who attach high or low importance to others’ welfare. A test of the participants’ civic knowledge and a measure of the political reputation they have in their classrooms will be used to investigate potential differences between the two groups. Finally, in view of expected gender differences in attaching importance to others’ welfare, gender will be controlled in the main analyses. Immigrant status also will be controlled, since values, attitudes and behaviors may differ between adolescents whose parents were born in the country and those whose parents were not. When significant gender and immigrant status differences appear, they will be reported in the running text.

## Method

### Participants

An age cohort of 16-year-old adolescents was followed in a longitudinal project concerned with the political socialization of young people. The study was conducted in a mid-sized city in Sweden from 2010 to 2015. The target sample was all the 1052 students listed in the student files of the city’s schools. The analytical sample, with data on the measures used comprised 857 persons (81%). The percentage of participants both of whose both parents were born outside Nordic countries was 18%, which is close to the national proportion. The city is like the country regarding its immigration rate, income, level of education, unemployment rate, and turnout in the last election (Statistics Sweden, 2010).

### Procedure

The cohort was recruited from three high schools, with a total of 57 classes, and strategically chosen to represent the social and demographic characteristics of the city’s adolescents. An opt-out procedure was employed where the youth’s parents were informed about the study before the first assessment and had the opportunity to refuse their children’s participation (less than 2% did). The school assessments took place in classrooms during regular hours. Trained test administrators distributed a self-report questionnaire to the participants without their teachers being present. A contribution of 1000 SEK (about 120 USD) was made to the class fund. One of the six local boards of Sweden’s National Ethics Review Board approved all procedures.

### Measures

The measures were developed in the project unless otherwise stated.

#### Political interest

This measure combines political interest with conscious positive feelings about politics (Stattin & Russo, in press). First, level of political interest was measured by asking: “How interested are you in politics?” (see the ANES (2014) Panel Study for the same item). The response options ranged from 1 (*totally uninterested*), to 5 (*very interested*). Second, the participants were asked: “People differ in how they feel about politics. What are your feelings?” Responses were on a six-item scale from 1 (*loathe)* to 6 (*great fun)*. The interest question was re-scaled to range between 1 and 6, and the two items were aggregated. The correlation between the two items was 0.59, *p* < 0.001.

#### Universalism

Universalism as a value has three components: tolerance, societal concern, and protecting nature. Multidimensional scaling studies have repeatedly confirmed the existence of societal concern and protecting nature as separate components, but this is less so with tolerance (see Schwartz et al., 2012, p. 668). Hence, societal concern and protecting nature are the two features of universalism attended to in the study. Societal concern is in this study’s terminology “attaching importance to other people’s welfare”. The participants were asked about the personal importance of each of these two basic characteristics of the value of universalism. After the stem question “How important is the following to you?” the adolescents were presented with a list of 21 value statements, of which 5 encompassed importance attached to other people’s welfare (“Equality, equal opportunities for all”, “A peaceful world free of war and conflict”, “Helping other people”, “Social justice, removing injustice and caring for the less fortunate in society”, and “Working together with others for a better society”)^.^ Second, three value statements addressed environmental values (“Respecting nature and thinking ecologically”, “Protecting the environment and preserving nature”, and “Preventing pollution of the environment and not wasting resources”). The response scale ranged from 1 (*not at all important*) to 5 (*very important)*. Alpha reliability was 0.80 for attaching high levels of importance to others’ welfare, and 0.89 for environmental values.

By using two scales, further aspects of having environmental concerns were examined: collective environmental efficacy and behavioral markers of environmental concerns.

#### Collective environmental efficacy

This was a measure of the participants’ experiences of being part of affecting climate change together with other people. The stem question was: “How well do the following apply to what you think?”, followed by two statements: “If we all pitch in, we can solve many environmental problems”, and “If we work together, we can do something about climate change”. The response scale ranged from 1 (*doesn’t apply at all)* to 5 (*applies very well)*. The correlations between the two items was 0.86.

#### Pro-environmental behaviors

Pro-environmental behaviors (Ojala, 2010) was measured on a scale containing 6 items, such as “Help my parents to sort at source”, “Use public transport, cycle or walk rather than being taken by car”, and “Turn off the light when I leave an empty room”. The responses ranged from 1 (*almost never)* to 5 (*almost always)*. Alpha reliability was 0.74.

Three examples of support for norms of democratic citizenship were measured: inclusive attitudes towards immigrants, positive attitudes towards democratic principles, and trust in social movements.

#### Inclusive attitudes towards immigrants

Inclusive attitudes towards immigrants were measured by 3 items with the statements: “Our culture is enriched when people from other countries move here”, “In the future, Sweden will be a country offering exciting encounters between people from different parts of the world”, and “Immigrants should have same rights as people born in Sweden”. The response scale ranged from 1 (*doesn’t apply at all*) to 5 (*applies very well*). Alpha reliability was 0.81.

#### Positive attitudes towards democratic principles, rights and responsibilities

These positive attitudes were measured by 6 items, based on the question “There are different views on how society should function. What is your view?”, followed by statements about democratic rights, “All citizens are free to choose their leaders”, “Women should have the same rights as men”, and “Everyone should have the right to publicly criticize the government”, and about democratic obligations, *“*You must follow the laws determined by the majority”, “Violence must never be used in political protests”, and *“*You have to pay taxes that are democratically determined”. The responses were from 1 (*disagree completely*) to 4 (*agree completely*). Alpha reliability was 0.73.

#### Trust in social movements

The participants were asked about their trust in “social movements (e.g., for human rights, the environment, or animal rights)?” on a scale from 1 (*no trust at all)* to 4 (*a lot of trust)*.

A factor analysis (principal axis extraction with varimax rotation) was used to examine the dimensionality of the variables covering environmental issues (environmental values, collective environmental efficacy, initiating discussions about environmental issues, and pro-environmental behaviors) and support for norms of democratic citizenship (inclusive attitudes towards immigrants, positive attitudes towards democratic principles, and trust in social movements). Two factors were expected, but only one was extracted, with factor loadings from 0.56 to 0.75. This factor was used in the comparison between the cluster groups.

The adolescents’ offline and online forms of political participation distinguished between collective and solitary activities.

#### Political activities offline during the last year

Offline political activities contained 5 examples of collective political activities, i.e., activities pursued with other people, “Worked voluntarily for a good cause”, “Attended a meeting concerned with political or societal issues”, “Collected signatures”, “Distributed leaflets with a political content” and “Took part in a legal demonstration or strike”, and 4 examples of expressing one’s political opinions alone, “Donated money to a good cause”, “Boycotted or bought certain products for political, ethical or environmental reasons”, “Donated money to support the work of a political group or organization”, and “Wore a badge or a t-shirt with a political message”. The response scale ranged from 1 (*no*) to 3 (*yes, several times*). The alpha reliabilities for these measures were 0.82 and 0.77 at T1 and T2.

#### Member of a political/civic association

The participants were asked if they were a member of any of three types of associations: Political association, Environmental association, or an Association for peace or human rights (e.g., the Peace Movement, Amnesty International).

#### Political activities on the internet during the last year

Political activities on Internet comprised 3 types of activities. Having political discussions with friends contained 4 items, “Discussed societal or political questions with friends on the internet”, “Linked news to my friends”, “Chatted with friends on the net about something I’ve seen on the news”, and “Sent friends music that I think has a good political and societal message”. Having political discussions with strangers was a single item, “Discussed societal issues with people I don’t know”. Solitary following of political and societal information contained 3 items, “Sought information about politics or societal issues on the internet”, “Read about politics in a blog”, and “Viewed videos or film clips about societal issues or politics”. The response scale ranged from 1 (*no*) to 3 (*yes, several times*). Alpha reliabilities for the first and third scales were 0.70 and 0.73, respectively.

#### Standby citizenship

Standby citizenship was measured on a scale containing 4 items. The stem question was “How active should people be on political issues? What do you think? For me it is important to: “Protest when people are treated unfairly”, “React when something important is at stake”, “Give up my time when others ask me to step in”, and “Keep an eye on what is going on, so that I can do something if needed”. The responses were from 1 (*not important at all*) to 4 (*very important*). Alpha reliability was 0.82.

To examine whether the expected two clusters of politically interested adolescents with high or low importance attached to others’ welfare had different political reputations and levels of civic knowledge, their political reputation among agemates and their knowledge of political and societal issues according to a knowledge test were examined.

#### Political reputation in class

All students in the classes in the ten schools were asked: “Here, different types of people are described. Which of your classmates are like these people? Two of the types described were “Keeps track of what is going on in Sweden and in the world” and “Often starts discussions about societal issues in class”. The students were asked to nominate three students in their classes who matched each of the two descriptions, self-nominations excluded. For classes with more than eight students, the number of times the students were nominated for each of the two descriptions was divided by the total number of students in the classes who participated in the data collection. The two items were aggregated. Alpha reliability was 0.87.

#### Civic knowledge

The knowledge test consisted of 8 questions, such as “Of which American state was the actor, Arnold Schwarzenegger, the governor?”, “Which of the following countries are not members of the EU?”, and “What does ‘development assistance’ mean?”.

Control variables were immigrant status and gender. Immigrant status was a dichotomous measure differentiating between students both of whose parents were born outside the Nordic countries (coded 1) and other students (coded 0). Gender was coded 0 = female, 1 = male.

### Plan of Analysis

In order to detect the proposed subgroups of adolescents, a hierarchical cluster analysis (Ward’s method) was used to identify the number of clusters for the two standardized measures of political interest and importance attached to other people’s welfare. The lower limit was set at 67% of the total error sums of squares explained to select the number of clusters (Bergman, Magnusson, & El Khouri, 2003). Next, as recommended by Kinder, Curtiss, & Kalichman, (1991), with knowledge of the number of clusters, a non-hierarchical cluster analysis, K-means clustering, was used to arrive at the final cluster solution. In order to avoid clusters with very high values, the two measures were recoded before the hierarchical cluster analysis. Standardized values above 2.5 were set at 2.5. Finally, ordinary ANOVAs were used to examine the clusters obtained on all the other measures. In all these analyses, F values were calculated after controlling for gender and immigrant status.

## Results

### Cluster Analysis

The hierarchical cluster analysis identified five clusters for the two measures of political interest and importance of other people’s welfare. The cluster solution explained 71% of the total error sums of squares. Descriptive information about these clusters, after subsequent K-means clustering, are shown in Table 1.

**Table 1:** A cluster analysis of the adolescents’ political interest and attached importance to other people’s welfare

	LL	LA	AA	HL	HH
Political interest	−1.18	−1.05	0.07	0.73	0.95
Importance of others’ welfare	−1.30	0.51	−0.03	−1.37	0.79
*N*	123	166	234	78	256
%	14.4	19.4	27.3	9.1	29.9
% boys^a^	66.7	39.2	41.5	74.4	44.9
% immigrants^b^	10.6	16.4	11.5	5.2	19.5

Low values (lowest to −0.60); Average values (> −0.60 to 0.60); High values (> 0.60)

^a^F (df = 4) = 48.88, *p* < 0.001

^b^F (df = 4) = 14.46, *p* = 0.006

Three clusters were identified with low or average values on both political interest and perceived importance of others’ welfare. Further, there were two clusters where the participants had high political interest. One of them, the HH group in Table 1, attached high importance to other people’s welfare, while the other, the HL group, attached low importance to other people’s welfare. In effect, the cluster analysis identified the two proposed subgroups as naturally occurring profiles for political interest and importance of other people’s welfare. Note that the label HH will be used for the adolescents with high political interest and high importance attached to other people’s welfare and HL for the adolescents with high political interest and low importance attached to other people’s welfare.

Based on previous research showing that societal concern is rated higher among females than males, it was expected that the HH cluster would include a higher percentage of females than males. This was not the case. The HH group contained roughly similar proportions of females and males (55 vs. 45%). Males, by contrast, were overrepresented in the HL group. Three of four of the adolescents in this group were males. This is a noteworthy finding. It suggests that the male overrepresentation of politically interested people that is commonly found may be partly accounted for by politically interested males who seem to attach low levels of importance to other people’s welfare. It should be added here when analyzed as original variables, political interest was unrelated to gender. Finally, immigrant status differed significantly between the clusters, with a higher than expected frequency in the HH cluster and a lower than expected frequency in the HL cluster.

### Correlations Among the Comparison Variables

Correlations among the comparison variables are reported in Table 2. To save space, the different manifestations of environmental values (environmental efficacy and pro-environmental behaviors) have been omitted. In addition, all varieties of political activities have been merged into two categories—offline and online activities. Inclusion of just these two types does not change the broader conclusions. Three features stand out. First, of all the variables in Table 2, standby citizenship was the variable that most systematically correlated with the other measures. Second, the political activities measures (offline and online political activities and membership of civic/political associations) correlated with each other, as was also the case for ecological values, inclusive attitudes towards immigrants, positive attitudes towards democracy, trust in social movements, and standby citizenship. Third, with regard to gender, girls scored higher than boys on environmental values, inclusive attitudes towards immigrants, positive attitudes towards democratic principles, trust in social movements, and standby citizenship. Boys scored higher on online political activities. Not surprisingly, immigrant adolescents scored higher on inclusive attitudes towards immigrants.

**Table 2:** Correlations among the study variables

	Attitudes immigrants	Attitudes democracy	Trust social m.	Political efficacy	Offline activities	Membership organization	Online activities	Standby citizen	Immigrant status	Gender
Environmental values	0.19***	0.22***	0.29***	0.15***	0.10**	0.02	0.08	0.23***	−0.01	−0.15***
Attitudes immigrants		0.36***	0.27***	0.23***	0.19***	0.12***	0.17***	0.41***	0.20***	−0.25***
Attitudes democracy			0.26***	0.21***	0.05	−0.01	0.06	0.30***	0.00	−0.12***
Trust social movements				0.21***	0.13***	0.09*	0.17***	0.38***	−0.03	−0.26***
Political efficacy					0.25***	0.19***	0.38***	0.35***	0.01	0.05
Offline activities						0.27***	0.38***	0.22***	0.08	−0.02
Membership organization							0.29***	0.13***	0.04	−0.00
Online activities								0.25***	0.08	0.15***
Standby citizen									−0.03	−0.19***
Immigrant status										0.02

*p < 0.05, **p < 0.01, ***p < 0.001

### Environmental Values, Attitudes and Behaviors

The five cluster groups were compared on environmental values, and on attitudes and behaviors related to environmental values, and on inclusive attitudes towards immigrants, attitudes towards democratic principles, and trust in social movements. It was expected that the HH adolescents would show high levels on these measures, whereas the opposite would apply to the HL group. As reported in Table 3, this seems to be the case.

**Table 3:** Differences between the five cluster groups on environmental values, attitudes towards immigrants, attitudes towards democratic principles, and trust in social movements

	LL	LA	AA	HL	HH	F	*p*	eta^2^	Cohen’s d
Environmental values	−0.91^a^	0.15^b^	0.08^b^	−0.79^a^	0.52^c^	68.32	<0.001	0.24	1.49
- Environmental efficacy	−0.59^a^	−0.22^b^	0.16^c^	−0.27^b^	0.43^d^	30.55	<0.001	0.12	0.78
- Pro-environmental behaviors	−0.57^a^	−0.25^b^	0.09^c^	−0.20^b^	0.40^d^	23.51	<0.001	0.10	1.73
Attitude immigrants	−0.70^a^	−0.14^b,c^	0.03^c^	−0.34^a^	0.49^d^	31.90	<0.001	0.12	0.84
Attitude democratic principles	−0.49^a^	−0.15^b^	0.02^b^	−0.17^b^	0.35^c^	15.77	<0.001	0.07	0.50
Trust in social movements	−0.60^a^	−0.25^b^	0.10^c^	−0.39^a,b^	0.48^d^	33.30	<0.001	0.13	0.60
Total (factor scores)	−1.00^a^	−0.21^c^	0.12^d^	−0.58^b^	0.66^e^	86.46	<0.001	0.27	1.38

All measures in the table are standardized. Across rows, superscripts represent significant differences (*p* < 0.05) between clusters in SNK post-hoc tests

Eta^2^ compares differences among all five groups, and Cohen’s d compares differences between the HH and the HL groups

For all three measures of environmental concerns, and attitudes towards immigrants, democratic principles, and trust in social movements, the HH group had significantly higher means than all the other groups. The adolescents in the HL group showed lower levels than the average means for the total sample. Of the five cluster groups, particularly low levels were found among the adolescents who had low levels of both political interest and importance to others’ welfare (the LL group). The effect sizes for all these measures when comparing the HH and the HL group ranged from medium (attitudes towards democratic principles) to very large (pro-environmental behaviors). At the bottom of Table 3 there is an analysis of the factor that captures all seven variables. The HH adolescents scored particularly high on this factor and the HL adolescents scored low. Cohen’s d was 1.38, which is a very strong effect size. Overall, these analyses confirm the hypothesis that politically interested adolescents have quite different environmental values, environmental behaviors and attitudes and attitudes towards immigrants, democratic principles, and trust in social movements according to whether they attach high or low importance to other people’s welfare.

For all measures in Table 3, gender was a significant variable at the 0.001 level. Females scored higher than males. Cohen’s d ranged from small (attitudes towards democratic principles) to medium effects (trust in social movements). The effect size for the factor was medium, 0.62

### Political Participation

The adolescents’ offline political activities, membership of political and/or civic associations, and their activities on the internet were examined. As reported in Table 4, the HH adolescents were significantly more involved in offline political activities, collective and solitary, and more engaged in civic/political associations than the adolescents in the other cluster groups. The effect sizes for the differences between the HH and the HL groups were small. The results were different for political activities on the internet, both collective and solitary, and political discussions with strangers on the internet. Here, both the HL and the HH adolescents were significantly more involved than the adolescents in the other three cluster groups. In short, offline political activities and memberships of civic/political associations were concentrated chiefly among the HH, whereas online activities were concentrated among both the HH and the HL. Significant gender differences were found for collective political activities on the internet and political discussions with strangers on the internet. Girls scored lower than boys. Collective activities offline were reported more by participants both of whose parents came from other countries. These gender and immigrant status differences, although significant at the 0.001 level, yielded low effect sizes.

**Table 4:** Differences between the five cluster groups on internal political efficacy, offline and online political activities, and membership of a civic or political association

	LL	LA	AA	HL	HH	F	*p*	eta^2^	Cohen’s d
Offline collective activities	−0.17^a^	−0.10^a^	−0.09^a^	−0.03^a^	0.24^b^	4.76	<0.001	0.02	0.26
Offline solitary activities	−0.29^a^	−0.23^a^	−0.07^a^	−0.08^a^	0.37^b^	13.02	<0.001	0.06	0.43
Civic/political association	−0.13^a^	−0.18^a^	−0.16^a^	−0.01^a^	0.34^b^	10.42	<0.001	0.05	0.27
Online collective activities	−0.36^a^	−0.36^a^	−0.07^b^	0.25^c^	0.39^c^	22.95	<0.001	0.10	0.13
Online solitary activities	−0.51^a^	−0.48^a^	−0.04^b^	0.41^c^	0.46^c^	39.43	<0.001	0.16	0.03
Online activities, strangers	−0.22^a^	−0.27^a^	−0.21^a^	0.38^b^	0.35^b^	17.04	<0.001	0.08	0.02

All measures in the table are standardized. Across rows, superscripts represent significant differences (*p* < 0.05) between clusters in SNK post-hoc tests

Eta^2^ compares differences among all five groups and Cohen’s d compares differences between the HH and the HL groups

### Standby Citizenship

To be a stand-by citizen, by being willing to act politically in the future if people were at risk of harm or treated unfairly, was compared between the five cluster groups. As shown in the upper part of Table 5, such willingness was particularly concentrated among the HH adolescents. Even after controlling for offline and online activities and memberships of civic and political associations, and the covariates, highly significant differences were found among the cluster groups, with a medium to high effect size. The effect size when comparing the HH and the HL groups was 0.95, which is a large effect. There were significant gender differences, with a low effect size (Cohen’s d is 0.40). Girls scored higher than boys on standby citizenship.

**Table 5:** Differences between the five cluster groups on a measure of standby citizenship, and results from an ANOVA controlling for membership of civic/political associations, offline and online political activities, and gender and immigrant status

	LL	LA	AA	HL	HH	F	*p*	eta^2^	Cohen’s d
Standby citizen	−0.76^a^	−0.33^b^	0.07^c^	−0.16^b^	0.57^d^	29.87	<0.001	0.11	0.95
			F	p	eta^2^				
Covariates									
Offline collective activities			0.06	0.802	0.00				
Offline solitary activities			10.46	0.001	0.01				
Civic/political association			0.99	0.321	0.00				
Online collective activities			5.17	0.023	0.00				
Online solitary activities			30.58	<0.001	0.03				
Online activities, strangers			5.63	0.018	0.01				
Immigrant status			2.38	0.123	0.00				
Gender			41.27	<0.001	0.04				

All measures in the table are standardized. The predicted means adjusted for the covariates were −0.60, −0.29, 0.05, −0.12, and 0.47. Across rows in the first column of the table, superscripts represent significant differences (*p* < 0.05) between clusters in SNK post-hoc tests

Eta^2^ compares differences among all five groups and Cohen’s d compares differences between the HH and the HL groups

### Political Reputation and Political/Societal Knowledge

What remains to be understood is whether the HH and HL adolescents differ in the way in which their political interest and knowledge are visible to others and in their knowledge of politics and society as revealed by an “objective” knowledge test. Information about the adolescents’ political reputations in their classes and their answers to a political/societal knowledge test were used. As reported in Table 6, both the HH and HL adolescents stood out as having a high political reputation and high political/societal knowledge compared with the adolescents in the other three cluster groups. In fact, the HL adolescents had significantly higher scores on the knowledge test than the HH adolescents. In short, in others’ eyes and as determined by their factual knowledge, the HH and the HL adolescents can be considered more politically sophisticated than the other adolescents. Gender differences appeared both for political reputation (F (1, 799) = 16.09, *p* < 0.001) and on the knowledge test (F (1, 799) = 16.45, *p* < 0.001). Boys had a higher level of political reputation and higher scores on the knowledge test than girls. A significant effect of immigration status was also found for the knowledge test (F (1, 799) = 21.10, *p* < 0.001), with participants whose both parents were born in other countries having lower scores than other participants. The effect sizes for the gender and immigrant-status effects were low.

**Table 6:** Differences between the five cluster groups on political reputation in class and a test of the adolescents’ political/societal knowledge

	LL	LA	AA	HL	HH	F	*p*	eta^2^
Political reputation	−0.22^ab^	−0.32^a^	−0.04^b^	0.39^c^	0.26^c^	13.38	<0.001	0.06
Civic knowledge	−0.18^ab^	−0.41^a^	−0.04^b^	0.48^d^	0.23^c^	14.49	<0.001	0.07

All measures in the table are standardized. Across rows, superscripts represent significant differences (*p* < 0.05) between clusters in SNK post-hoc tests

Eta^2^ compares differences among all five groups and Cohen’s d compares differences between the HH and the HL groups

## Discussion

Politically interested citizens are more knowledgeable, more interested in engaging in political and civic activities, talk more often about political and civic issues with people around them, and more prepared to be mobilized than other citizens (Prior, 2019). But the common tribute paid in democratic societies to citizens’ interest and their critical voice in political processes needs to be qualified. Politically interested young citizens are influential but they are not a homogenous group. Schwartz’s theory of basic human values (Schwartz et al., 2012), and specifically the value of attending to others’ welfare, was used in this study to identify and explain the diverse attitudes and behaviors of politically interested adolescents.

A cluster analysis of 16-year-olds’ political interest and the importance they attached to others’ welfare identified five groups, including ‘high political interest and *high* importance attached to others’ welfare’ and ‘high political interest and *low* importance attached to others’ welfare’. Of these two groups, the adolescents with high political interest and high levels of importance attached to others’ welfare scored high on environmental values, and had inclusive attitudes towards immigrants, high evaluations of democratic principles, and high trust in social movements. Effect sizes when comparing these two groups were for the most part large. Comparisons of their political activities revealed that the adolescents with high political interest and high levels of importance attached to others’ welfare were also more engaged in political activities offline and were members of civic/political associations to a greater extent. Effect sizes were small. Further, the adolescents with high political interest and high levels of importance attached to others’ welfare reported themselves to be more ready to step in if something jeopardized the welfare of others. The effect size was large. The conclusion is that highly politically interested adolescents have very different values, attitudes, and engagements in political activities according to whether they attach high or low importance to others’ welfare. Highly politically interested adolescents are apparently not a uniform group. In one respect, however, they were found to be similar. They both engaged in political activities online more than the other cluster groups of adolescents.

It is well-known that a concern for the welfare of others is strongly correlated with environmental values (Cieciuch & Schwartz, 2012). This was also true in the present sample (*r* = 0.60, *p* < 0.001). A high correlation between these basic value dimensions is likely to be present in all conceivable subgroups, such as adolescents with high political interest who attach high importance to others’ welfare and politically interested adolescents who attach low importance to others’ welfare (*r* = 0.54 and 0.44 respectively, in this study). However, what would not be immediately predicted is that members of the former subgroup of adolescents, in comparison with the latter, have high evaluations of democratic principles, inclusive attitudes towards immigrants and high trust in social movements, are more often involved in offline political activities, are more often members of political/civil associations, and more likely to report themselves as standby citizens. This covers the range of differences predicted in the study. Future studies are needed to understand the combined role of political interest and importance of others’ welfare for adolescents’ political attitudes and behaviors long-term.

An explanation for why politically interested adolescents with high or low importance attached to others’ welfare differ in their political values and attitudes could be that the two groups may contrast in their political knowledge and political visibility to others. This, however, does not seem to be the case. The two groups of adolescents had about the same political reputation in their classes, and they achieved about the same results on a knowledge test of societal issues. Both groups differed significantly from the other groups of adolescents in these regards. Given their political reputation and documented political knowledge, they might have been expected to be politically active offline to the same or greater extent than the other groups. But the politically interested adolescents attaching low importance to others’ welfare were less politically active offline. This finding emphasizes the fundamental asset to society constituted by politically interested adolescents with high importance attached to others’ welfare. The mean level of political activity offline for politically interested adolescents with low importance attached to others’ welfare was, on these measures, at the same level as that of the average person in the sample.

Broad, general values, like valuing human dignity and human rights, have been said to underpin citizens’ political participation (Barrett, 2016), but the research linking values to political participation has mainly focused specifically on political aspects, such as left/right, libertarian/authoritarian, traditional, postmaterialist, and democratic (for an overview, see Heath, Lindsay & Jungblut, 2022). These are narrower values, which are assumed to be more directly linked to political action than the global values in Schwartz’s theory of basic human values (Schwartz et al., 2012). Still, the present findings that a global value like attaching importance to other people’s welfare has strong implications for politically interested young people’s attitudes, behaviors, political action offline, and preparedness to act under certain types of circumstances in the future is striking. Apparently, the importance attached to other people’s welfare has high explanatory power in shaping adolescents’ political values, attitudes and activities and preparedness for future political action. One practical implication of this may relate to the processes through which adolescents become recruited to political activities. What the present results suggest is that a conscious strategy in mobilizing adolescents to political action, such as volunteering, is to provide value-based motivations that connect young people’s high political interest to their genuine care for other people. This conclusion is sustained by the observations that willingness to step in if others’ welfare was at stake, the standby citizenship construct, was correlated with the measures of political action, environmental values, attitudes towards immigrants and democratic principles, and trust in social movements, all to a substantial extent, and that being a standby citizen was concentrated solely among the highly politically interested adolescents attaching high levels of importance to others’ welfare.

This study showed a roughly equal proportion of males and females across the two groups with high political interest. What was most noteworthy, and not predicted, was that the group of adolescents with high political interest and attaching low importance to the welfare of others mainly contained males (74%). The overrepresentation of highly politically interested males found in previous studies may hide the fact that some of the highly interested males do not channel their political interest into common political activities or into a preparedness for future political activities. In addition to being heavily overrepresented by males, the group of highly politically interested adolescents attaching low importance to the welfare of others stand out in several respects. They are known to their classmates as having high knowledge of political issues and are interested in discussing political matters in class, but they do not seem to transfer their political interest into civic/political associations or into political activities with others offline. Further, they do not communicate their opinions in public, by, for example, donating to political or civic initiatives or refusing to buy products for political or other reasons, and they are not willing to act politically if people are in jeopardy. They seem to be content with having the internet and social media as their sources and modes of political activity. In line with its supposed opportunities, the internet seems to offer the politically interested who attach low importance to the welfare of others an appropriate arena for being an active citizen without requiring personal identification in exchange (Schuster, 2013).

This study could, in some sense, have been a study of differences between females’ and males’ values and political attitudes. High importance attached to others’ welfare was more common among females than males (*r* = 0.25, *p* < 0.001), while political interest was unrelated to gender. Females also scored higher than males on environmental values. Thus, females reported higher levels than males for the two values that make up universalism in Schwartz’s model – societal and environmental concerns. The gender differences reported in the current study are similar to those found in research on Schwartz’s basic values (Aredenghi et al., 2021; Döring et al., 2015; Schwartz & Rubel, 2005). Further, in line with higher environmental values, correlational analyses in this study showed that females had higher levels of pro-environmental behaviors, and had higher collective environmental efficacy than males.

In addition to environmental values, several other differences found between girls and boys go in the same direction as the differences found between the two groups of adolescents with high and low importance attached to others’ welfare. Girls scored higher than boys on inclusive attitudes towards immigrants, positive attitudes towards democratic principles, trust in social movements, and standby citizenship; as did the participants with high political interest and high attached importance to others’ welfare. Overall, the higher levels for girls than boys on the universalism value and measures of support for democratic norms – where these measures make up one common factor – can be interpreted in terms of gender socialization, a stronger other orientation and social responsibility among females (Zelenzny et al., 2000). Of course, other frames for interpretation are likely.

The similarities in outcomes between the two cluster groups and between females and males end here. There were no gender differences for offline political activities. In line with much previous research, females were less engaged in online activities such as talking to friends and strangers, and they had generally lower scores on the knowledge test (Kittilson & Schwindt-Bayer, 2012; Dassonneville & McAllister, 2018) as well as lower political reputation in their classes. The females´ hesitancy in stepping forward as political actors may reflect their general lack of access to supportive networks (Pfanzelt & Spies, 2019). Moreover, the females’ avoidance of online activities can be understood in the light of their overall tendency to hold back when faced political dissension (Djupe, McClurg, & Sokhey, 2018), but also has the consequence that females’ political engagements offline tend to make them more invisible to those who choose online activism as their political platform (Schuster, 2013).

There are potentially three lessons to learn from applying the results. First, high political interest on the part of young citizens is not per se necessarily an asset for welfare and democratic principles. Second, for people involved in recruiting volunteers to civic and political organizations and campaigns, the study underlines the importance of stressing motivations connected to basic human values, such as helping others, equality, justice, and giving something back to society (Gage & Thapa, (2012); Chambré, 2020). Third, for educators in general and civic educators in particular, what is required is a human-values perspective on adolescents´ citizenship, as recently discussed by Veugelers, (2021).

Numerous studies in the literature have linked political interest to a wide variety of political behaviors. In these studies, no differentiation was made within the group of persons who had a high political interest. Researchers generally concluded that political interest makes democracy work (Prior, 2010, p. 747). Potentially, this is the first study to attempt to identify and explain the different political attitudes and behaviors among highly politically interested young people who are highly influential by virtue of their political knowledge and ability to communicate it to others.

A main strength of this study lies in its identification of the broader values of highly politically interested adolescents that seem to make some of them more politically active offline than other politically interested adolescents. The findings are robust and seem to apply to a variety of types of offline political activities and involvement in civic and political associations. They show that offline political participation is primarily concentrated among highly politically interested adolescents who attach high importance to others’ welfare but that online political activity is equally common among highly interested adolescents who attach high and low importance to others’ welfare.

There are limitations that need to be mentioned. First, this is a cross-sectional study, which rules out the attribution of causality. Second, with a few exceptions, its measures were based on self-reports, which raises concerns about shared method variance. This is largely unavoidable, though, because the study focuses on adolescents’ perceptions of a key universalism value, societal concern, and how this value extends to other values, attitudes and behaviors, and political actions. Nevertheless, the problem of shared method variance should be acknowledged. Third, the analyses concerning engagement in political activities among members of the clusters did not consider individual differences in opportunities available for carrying out political actions or in the broader structural factors that promote or hinder people’s political activism (Meyer & Minkoff, 2004).

Fourth, the study is embedded in its historical, social, and cultural context. It was conducted in Sweden and included adolescents from a single Swedish city. The sample is broadly representative of the country as a whole (proportion of immigrants, income, level of education, unemployment rate, and voting turnout). That said, the study needs to be cross-validated in other countries with different political landscapes. Finally, it is a limitation that the study cannot empirically differentiate attached importance to the welfare of *all* other people, universalism, from the welfare of ingroup members, benevolence.

## Conclusion

Previous research has largely ignored the possibility that a high political interest may very well accommodate different basic values. In a cluster analysis of a Swedish mid-adolescent sample, it turned out that politically interested adolescents who attached high or low importance to others’ welfare appeared as two naturally occurring clusters. Although both had a high political reputation among their classmates and both scored high on a test of civic knowledge, the group of adolescents who attached high importance to others´ welfare showed substantially higher environmental concern, more inclusive attitudes towards immigrants, more positive attitudes towards democratic principles, and higher trust in social movements. They also turned up as characterizing stand-by citizens, willing to act politically in the future if people are at risk of harm or treated unfairly. They might not be politically active at the moment but are prepared to act if their basic values are threatened. Their participatory latency may be seen as a societal asset. However, whether they really will step in, if the time comes, forcefully enough either to block a political decision they disapprove of or, more generally, to halt ongoing global autocratization, is another question (Hellmeier et al., 2021). Noteworthy is that the politically interested participants who attached low importance to others’ welfare largely consisted of males.
